# An Alternative Molecular View of Evolution: How DNA was Altered over Geological Time

**DOI:** 10.3390/molecules25215081

**Published:** 2020-11-02

**Authors:** Fredric M. Menger

**Affiliations:** Department of Chemistry, Emory University, Atlanta, GA 30322, USA; menger@emory.edu

**Keywords:** preassembly, neo-Darwinism, human intelligence, cat domesticity, Cambrian explosion, convergent evolution, non-coding DNA

## Abstract

Four natural phenomena are cited for their defiance of conventional neo-Darwinian analysis: human intelligence; cat domesticity; the Cambrian explosion; and convergent evolution. 1. Humans are now far more intelligent than needed in their hunting–gathering days >10,000 years ago. 2. Domestic cats evolved from wildcats via major genetic and physical changes, all occurring in less than 12,000 years. 3. The Cambrian explosion refers to the remarkable expansion of species that mystifies evolutionists, as there is a total lack of fossil evidence for precursors of this abundant new life. 4. Convergent evolution often involves formation of complex, multigene traits in two or more species that have no common ancestor. These four evolutionary riddles are discussed in terms of a proposed “preassembly” mechanism in which genes and gene precursors are collected silently and randomly over extensive time periods within huge non-coding sections of DNA. This is followed by epigenetic release of the genes, when the environment so allows, and by natural selection. In neo-Darwinism, macroevolution of complex traits involves multiple mutation/selections, with each of the resulting intermediates being more favorable to the species than the previous one. Preassembly, in contrast, invokes natural selection only after a partially or fully formed trait is already in place. Preassembly does not supplant neo-Darwinism but, instead, supplements neo-Darwinism in those important instances where the classical theory is wanting.

## 1. Introduction

Current evolutionary theory is based on neo-Darwinism. A key component of neo-Darwinism, mutation, is accepted as a main source of variation in the evolutionary mechanism. For example, when a colony of bacteria is exposed to penicillin, all the bacteria might be killed except for one that happens to have, via a mutation, a new enzyme (called penicillinase) that is able to deactivate the penicillin. A lone bacteria, thriving despite the presence of penicillin, expands into a newly evolved colony of penicillin-resistant bacteria. Demise of all bacteria except the lone mutant exemplifies Darwin’s concept of “natural selection”. This is neo-Darwinism in action: one new mutation; one new enzyme; and one new competence, all dispersed throughout the colony to embody what is known as “microevolution” [1]. The validity of the construct, one of the great ideas in biology, is beyond doubt.

Macro-evolution (the development of complex traits, novel morphologies, and new species via multi-gene modifications) is far more difficult to understand. In the present paper, four important examples of macro-evolution are discussed that neo-Darwinism fails to adequately address: (1) Human intelligence. Humans are now more intellectually capable than can be rationalized by current evolutionary theory (unless the living-off-the-land activities of our hunter-gatherer forefathers >10,000 years ago are connected to solving differential equations, writing symphonies, designing computers, or even driving an automobile). (2) Cat domesticity. Domestication of wildcats into housecats, a process involving multiple genetic and physical modifications, took place in less than 12,000 years. This is a trivial time period relative to both mutational and evolutionary time-scales, making it difficult to invoke neo-Darwinism as the source of wildcat-to-housecat conversion. (3) The Cambrian explosion. The Cambrian period began about 540 million years ago, and during this period an incredible diversity of life was created. In fact, it was the time when most of the currently known body-plans first emerged. Yet fossils of ancestors (“precursors”) to Cambrian life cannot be found, in contrast to what one expects from neo-Darwinism. S. J. Gould once commented that: “The Cambrian explosion was the most remarkable and puzzling event in the history of life” [2]. (4) Convergent evolution. Bats and whales both evolved an extremely complicated, multi-organ echolocation system for catching prey. Since the two animals lack a common ancestor, their echolocation abilities must have evolved independently. Although mutations are rare, random, and mainly harmful, they managed to find their way through a maze of biological complexity, with the aid of natural selection, not once but twice.

An alternative model that clarifies all four evolutionary puzzles, designated “preassembly”, will be set forth momentarily. Note that preassembly is meant to supplement, not supplant, neo-Darwinism. As already mentioned, neo-Darwinism works well with microevolution and with those instances of macroevolution where step-by-step fossil evidence is available. However, gaps in our understanding of evolution, such as the four just cited, should not be sloughed off as minor “anomalies” or “aberrations”. Collectively, they arouse significant questions about the comprehensiveness of neo-Darwinism.

Theological concepts of evolution, incidentally, will not be taken up here since they belong to a separate domain of human thought.

### 1.1. Human Intelligence

Although the evolution of human intelligence has been discussed by me previously [3], it will be reviewed here briefly to support the three other additional examples of preassembly. A main problem for neo-Darwinism centers on the following question: How can a large energy-costly set of genes, each member of which probably had little apparent benefit when first created individually, all gather into a permanent existence in the genomes of each and every member of a small population (that was dispersed and geographically isolated over a huge planet) who had a low reproductive output, a low rate of beneficial mutations, and a low level of genetic contact [3]? Supporters of neo-Darwinism have yet to explain just how mutations favorable to intelligence (and created individually often in remote parts of the world) assembled into each and every human genome. A gathering of these genes seems to have been too pervasive, too “perfect” to be dismissed as the product of, for example, wars and migration. However, an even more important point arises. If our ancient ancestors owned a modern but largely underutilized intelligence, then a neo-Darwinian explanation for intelligence fails because natural selection would disfavor such energy-expensive and inactive genes [4]. If, on the other hand, abstract thinking in humans evolved only in more recent times, then neo-Darwinism must be side-stepped with equal conviction. This is because organs of complexity, such as the brain, are claimed to have evolved in tiny steps over excruciatingly long time periods [5,6]. Evolution via preassembly skirts these difficulties, as will be now shown.

It is proposed that when *Homo erectus* departed Africa and expanded into Europe and Asia, humans were already endowed with a host of intelligence-related genes (“preassembly”). These genes were stored, in a masked state, within the huge non-coding portion of human DNA. In humans, 98% of the DNA is non-coding, with only 2% of the DNA needed for our ca. 20,000 genes [7,8]. In principle, therefore, the non-coding portion of the DNA has the potential of accommodating almost a million inactive genes. Genes that are silenced and stored among this vast library of genetic material could continue to mutate further over extensive time periods dating, perhaps, from the beginnings of biology. In other words, non-coding DNA libraries serve as a repository, as a way-station, for evolutionary developments lying in the distant future. 

Five other important features of preassembly should be mentioned: (1) Precursors of intelligence genes, being silenced when stored, cost little by way of energy and therefore survived rapid elimination that would often accompany expensive but unused traits [9]. (2) The presence of any particular masked gene, or gene precursor, in humans’ early DNA library was entirely fortuitous. So was the unguided gathering of the genes needed for a complex trait, a fact mitigated by the extremely long times relegated to gene storage. One cannot fault preassembly for having a fortuitous component given that neo-Darwinism is also predicated on fortuity. In this sense, preassembly and neo-Darwinism overlap. (3) It is not claimed that these silent mutations could foresee their destiny, i.e. that they could anticipate how they would eventually perform. They were simply buried among a vast number of other non-coding genes (sometimes incorrectly referred to as “junk DNA”) awaiting release. (4) The non-coding genes in question might possibly have been useful, in some measure, to their host cell, thereby enhancing the genes’ survival chances [10]. (5) It is also possible that the library included what might be called “proto-genes”, i.e., genes that coded for proteins, or protein segments, not yet fully operative and thus requiring additional modification. Even “proto-genes”, however, could have been valuable in hastening macroevolutionary timespans.

I have proposed that as *Homo erectus* evolved into *Homo sapiens*, masked intelligence genes were being continuously unmasked. In other words, humans were taking more and more advantage of their long-held, but underutilized, mental capacity. The proposal is based on epigenetics defined as changes in organisms caused by the modification of gene expression rather than by alteration of the genetic code itself [11]. Environmental conditions (temperature, humidity, salinity, and diet) and biochemically-related factors (fear, hunger, and stress) are all possible non-mutational effectors of epigenetics. In the case of human intelligence, I have set forth the idea that the brain itself is the motivating force behind the release of buried intelligence genes in a kind of “cranial feedback” mechanism [3]. Accordingly, as prehistoric man developed mental acuteness, through problem-solving in the course of everyday living, there were concurrent releases of brain messengers that activated heritable genes engaged in brain action. By this means intelligence was passed on to the next generation. Without such a cranial feedback system, intellectual improvements unrelated to mutational events would be confined to only one generation. This neo-Lamarckian view implies that the brain’s chemical output is epigenetically connected to cellular genetics, a concept that has received experimental support [12,13,14]. The model is also consistent with a human-level intellect being found throughout the world (recall the neo-Darwinian difficulty of assembling individual intelligence genes spread among widely disparate populations). Moreover, assuming that masked intelligence genes still lurk in our non-coding DNA, the model predicts a continual growth in the population’s average intelligence [15]. This could arise, in part, from gene-activating effects of our educational systems and everyday mental challenges. For further details of this key example of preassembly in biology, consult the paper entitled “Molecular Lamarckism. On the Evolution of Human Intelligence” [3].

### 1.2. Cat Domesticity

Domestication of cats from wildcats embodies a suite of new traits including the appearance of (a) a reduced level of fear resulting in an increased tolerance of human beings; (b) a social behavior during domestication, but a solitary one in the wild (unique in the cat family); (c) a polyestrous reproduction; (d) a lovable vocalization; (e) lengthened intestines; (f) improved long-term memory; and (g) a 30% smaller brain size. All these traits appeared within the last 4000–12,000 years (opinions vary), far too short a time relative to that usually relegated to the evolution of complex, multigene, macroevolutionary events [16]. The neo-Darwinism mechanism is replete with sequential single mutations, each one being usually screened by natural selection prior to the appearance of the next mutation. This excruciatingly slow process, especially in mammals (where horse evolution from dog-sized animals took 50 million years [17]), simply cannot account for the genetic and physical innovations within domestic cats appearing in a mere 12,000 years [18].

The most obvious explanation for the wildcat-to-housecat conversion is that silenced genes for housecats had been already preassembled in the wildcat’s genome. If this is true, then one needs to speculate as to how these genes became rapidly unmasked. Darwin proposes in his discussion of the “domestication syndrome” that becoming accustomed to human food rewards is most likely the major force that had altered the cats [19]. However, a diet of what was likely rats in human refuse piles is not classically mutagenic and, in any case, Darwin did not know about mutations. However, a recent comparative analysis of the house-cat genome also suggests that becoming accustomed to the human environment did indeed promote domesticity [20,21]. Preassembly lends strength to this conclusion but, in addition, also provides a theory for just how such gene awakening happened. Thus, a neo-Lamarckian preassembly mechanism is proposed in which wildcats became exposed to the human environment with favorable attributes such as a reduced stress level. In a trivial number of years, repressed domesticity genes, already in place within the non-coding portion of the wildcat genome, awakened throughout the species. Additionally, in this manner did the domestic cat appear on the scene at such a remarkable rate.

By invoking virtually unlimited time-spans one might “justify” mostly any evolutionary story, however improbable. Indeed, it was precisely to avoid this common subterfuge that a complex evolutionary event occurring in less than 12,000 years was selected for the present discussion. Restricted to these few thousand years, neo-Darwinian transformation, invoking the creation of multiple, slowly attained and screened mutations, defies plausibility. Contributing to the improbability, these mutations all had to be devoted to a common function: domesticity. Additionally, since the mutated genes would have appeared individually in different cats, gene-gathering into single genomes only adds to the neo-Darwinian’s prodigious time-costs. Preassembly, on the other hand, need not be concerned about highly coordinated, low-probability mutations, followed by slow gene dispersal, all occurring in a whisper of time. This is because many of the genetic necessities had already been created far back into the past history of the wildcat, in accordance with the tenets of preassembly.

### 1.3. The Cambrian Explosion

About 540 million years ago the Cambrian period began, and within 5–15 million years an incredible diversity of life was created [22,23]. Large numbers and varieties of arthropods were suddenly plentiful in the Cambrian seas. These invertebrates, with their exoskeletons and segmented bodies, included insects, spiders, shrimp, termites, lobsters, millipedes, and the most well-known Cambrian occupant, the trilobite [24]. A worldwide spread of earliest plankton, worms, mollusks, shelled protozoans, and sponges also emerged [25]. In addition, chordates, animals with a dorsal nerve cord, have been found in Cambrian fossils [26]. Most of the animal body-plans known today materialized in what has become known as the “Cambrian explosion”. Additionally, here is the most incredible fact: precursors of all these abundant creatures are not to be found [27]. That is to say, fossils in older rock below the Cambrian layer lack expected ancestors of Cambrian life. What is probably the most dramatic event in evolution still eludes explanation. Neo-Darwinism demands precursors, but they are not there, pure and simple. Modern biology has been to this very day confronted by a serious problem.

The Ediacaran period immediately preceded the Cambrian for a hundred million years. Fossils from the Ediacaran reveal that life was comprised mainly of bacteria and algae along with organisms that were at once multicellular, marine, soft-bodied, rounded in form, and immobile while organized into colonies shaped as tubes, discs, bags, and fans [28]. During their stationary existence on a muddy sea-bed, they presumably absorbed nutrients through their skins. These organisms, none of which resemble any living creature, eventually became extinct. Reasons for the absence of fossil evidence supporting the presence of more complex forms, i.e., the precursors of Cambrian life, have been proposed. Among these the most common is that precursors were soft-bodied and did not fossilize well. Countering this speculation, however, is the fact that lower Ediacaran animals were also soft-bodied, yet they fossilized readily. As will be now seen, the preassembly mechanism for evolution finally provides a solution to the perplexing Cambrian conundrum.

A possible reason that precursors to Cambrian life have not been found is that they never existed in the first place. Preassembly proposes that during the 100 million years prior to the explosion (i.e., the entire Ediacaran era) genes or proto-genes potentially useful for creation of Cambrian fauna were first formed, then silenced if necessary, and finally stored among a vast library of other non-coding genes. When the Cambrian era arrived, accompanied by environmental changes likely including lowered temperatures, the protected genes were released quickly and concurrently, leading to an “explosion” of new species without precursors. This mechanism clearly imparts plausibility to the rapid but intermediate-deficient Cambrian explosion.

The preassembly model leads to the question of whether it is reasonable that genes, in a silenced form and residing among other non-coding DNA, could actually survive over millions of Ediacaran years while accumulating mutations and awaiting release. Relevant to this question is the observation that humans, rats, and mice all have 481 DNA segments of 200 bp, which are 100% conserved (no insertions or deletions). In addition, 5000 sequences of >100 bp are also ultraconserved among the three mammals [29]. Obviously, DNA can survive evolutionary time-scales, and thus the proposed persistence of stored Ediacaran genetic material until its Cambrian release is not out of line.

Darwin considered the appearance of trilobites with no apparent antecedent a problem of the “gravest nature” [30]. He ultimately resigned himself to the fact that he could not provide a satisfactory response to the quandary. To this day no one since Darwin, even though armed with modern genetics, has offered a convincing explanation of the Cambrian explosion. Preassembly of genes and gene precursors, despite uncertainties connected with it, is a step toward a fuller understanding of evolution. Note again that this is not to dispute the natural selection portion of neo-Darwinism. It is only its mechanism of change prior to selection that is open to question, but that same uncertainty happens to be critical to the formulation of a viable evolutionary theory.

Gould and Eldredge devised the term “punctuated equilibrium” when referring to evolution’s short bursts of intense speciation followed by lengthy periods of stasis or equilibrium [31]. The Cambrian explosion in the midst of relative inactivity is a prime example. Spurts of evolution are a curious phenomenon given that mutational forces, such a cosmic rays and radioactivity, are generally more constant in magnitude. Of course, evolutionary progress could no doubt have been stimulated when, for example, Earth changed in temperature or when the sea water increased its calcium or oxygen content. Rising sea oxygen levels, originating from photosynthetic cyanobacteria, have in fact been proposed as the trigger for the Cambrian explosion [32]. Even today this is a contentious assertion. Without entering into the debate, let it be said that a more favorable environment should not be presented as a mechanism for evolution. It merely sets the stage that allows evolution to occur by genetic factors of the type under consideration in this paper.

In summary, rapid and abrupt evolution in the Cambrian explosion of life is compatible with fast epigenic release of preassembled genes. Few intermediates are expected from this mechanism because entire ensembles of a trait’s genes or proto-genes are released. In other words, achieving the multiple genes needed for many complex traits occurs not via an excruciatingly slow, stepwise mutational sequence, followed by selective culling at each step, but by an epigenetic release long after the genes or proto-genes have been formed by mutation in a vast library of mainly irrelevant genetic material. Thus, the lack of intermediates in the Cambrian explosion, a momentous episode in the story of life, need no longer be dismissed as a “mystery”. No longer is it necessary to postulate that species ascended exclusively over long time-periods from closely related forms via insensibly fine gradations and intermediates that, in the case of the Cambrian era, probably never existed.

### 1.4. Convergent Evolution

Multiple examples are found in nature of two species having nearly identical complex traits that are not derived from a common ancestor. Thus, a so-called Tasmanian wolf strongly resembles a dog, although one is a marsupial and the other a mammal. Shark and dolphins have similar body designs although they are not related by millions of years. The panda is related to bears, whereas the red panda is related to raccoons. Plethodontid salamanders and chameleons both evolved long projectile tongues with which to catch insects. Unrelated fish species have a large spot on their tails to, presumably, fool prey into being uncertain as to the direction the fish is swimming. An additional example of such a convergent evolution, involving a complex sensing organ, will now be described in greater detail and shown to comply with preassembly theory.

Bats and toothed whales (that include dolphins) have developed an exquisite ability to hunt using echolocation, a type of biological sonar [33]. Other animals, e.g., rats [34], have far less sophisticated forms of echolocation. When a supersonic signal is emitted toward a prey, the resulting echo reveals the location of the prey. Bats and whales differ hugely in size; live in totally different environments; and lack a common ancestor. Yet they share the amazing convergent evolution of a multigene, multiorgan specialization. The following details of bat echolocation demonstrate a sophisticated trait whose capabilities are possessed by two species without any common ancestor [35,36,37,38].

Some bats use their larynx to produce ultrasonic waves that are emitted through the mouth or nose. Other bats produce clicks with their tongues. Sound is projected by a wrinkled fleshy nose that acts as a type of megaphone. The delay and frequency of sound waves bouncing back from objects hit by the sound waves inform the bat as to the speed, direction, size, and distance of the prey. A piece of skin in the front of the ear canal, the tragus, directs sound into the ear. Just before a bat emits a sound, tiny bones in the inner ear separate so that the bat’s hearing is not damaged. Once the larynx muscle has contracted, the middle ear relaxes, allowing the bat to hear incoming echoes. To help avoid deafening themselves on emitted sounds that can reach a remarkable 120 decibels, bat signals fall outside the bat’s audible frequency range. This works well because echoes have a lower frequency detectable by the bat. A single echolocation call can last anywhere from 0.2 to 100 ms in duration depending on the proximity of the prey [39]. The time intervals between subsequent calls is typically 100 ms for a bat searching for insects, but can decrease to 5 ms in the final moments of capture. The auditory systems of bats are particularly amazing. The key organ is the cochlea, a hollow spiral-shaped bone in the inner ear that converts sound vibrations into nerve impulses with the aid of hair cells. A specialized basilar membrane within the cochlea responds to the frequency of returning echoes. For example, the basilar membrane of the horseshoe bat has a disproportionate lengthening and thickening that responds to 83 kHz, the common frequency of the bat’s echo [40]. Bat ears can detect even tiny frequency modifications created by the echoes. Sensitivities of bat echolocation also rely on an important protein called prestin. Prestin is a type of biological motor functioning at microsecond rates, orders of magnitude faster than any other cellular motor protein. Information processing in the auditory cortex of the echolocator’s brains is also highly specialized in a manner not found in non-echolocating animals [41,42]. There are even “delay-tuned” neurons in the cortex that respond to a specific time-delay between the call and echo. Other neurons, called combination-sensitive neurons, respond to specific combinations of frequency and timing. Among the dozens of genes known to be involved in the auditory perception system, twelve are engaged in bone formation and are thought to enable bats to hear high frequencies.

Whales have also evolved a sophisticated echolocation system [43,44]. As expected from preassembly, bats and whales share many similarities. For example, prestin proteins resemble one another among diverse and unrelated echolocators. Moreover, genes that regulate development of the cochlear ganglion in bats and whales possess convergent substitutions. Of course, the needs of whales and bats differ widely, and abilities peculiar to the species are the result. For example, a sperm whale can echolocate prey 500 m away, whereas a bat’s echolocation distance is only 2–10 m. Such specializations may have evolved classically once the basic ear/brain structure, formed via preassembly, emerged from a common store-house of non-coding genes. One must always consider the possibility that preassembly and neo-Darwinism overlap to a certain extent. The important point is that similarities between complex convergent traits often correspond more to a preassembly, unguided heritage than to a mechanism of coincidental incrementalism.

The great virtue of preassembly is that, as we have just seen, it explains important events in nature that neo-Darwinism fails to do. This does not mean that the preassembly theory is without uncertainties itself. Issues with preassembly that still need clarification are mainly related to its epigenetic component. By what mechanism do masked genes for a trait, lying silently in wait within a huge array of non-coding genes, become selectively demasked? Once epigenetics becomes a more fully understood discipline, such questions should become less of an obstacle. Indeed, it should be realized that evolution is in fact a highly complex process that falls far outside the reach of DNA. 

Ideally, biologists would like to calculate evolutionary odds. For example: What are the odds that an echolocation system forms via neo-Darwinian step-by-step mutations? Answering such questions is now an impossible task, and will likely always be so. Even if mutational rates are assumed, the success rate of screening each mutation by natural selection (among many other components of evolution) cannot be predicted. It is for this reason that most arguments in this paper are non-quantitative, e.g., “Large numbers and varieties of arthropods were suddenly plentiful in the Cambrian seas”. Fortunately, qualitative statements such as this usually suffice to drive home most of the points. A quote from Nobelists Woodward and Hoffmann, taken from their classic paper, is relevant to the subject: “If the simple argument is true, then any approximate methods, as well as the now inaccessible exact solution, must obey it [45]”.

## 2. Conclusions

Preassembly helps in understanding the presence of human intelligence, cat domesticity, the Cambrian explosion, and echolocation. The converse is also true: human intelligence, cat domesticity, the Cambrian explosion, and echolocation provide evidence-supporting preassembly. The construct deserves attention because neo-Darwinism, with all its power to explain microevolution, does not deal well with many important features of macroevolution. Preassembly is thus attractive, if for no other reason than offering a supplementary view of evolution based on known biological processes. Accordingly, formation of genes for complex, multigene traits, exploited extensive residence times available to them as inert non-coding genes. An epigenetic (neo-Lamarckian) release led to partially or fully formed traits that were subsequently screened by natural selection. Both neo-Darwinism and preassembly depend upon fortuitous genetic events, but the timing of these events is obviously very different. Neo-Darwinism is a tedious, piecemeal macroevolutionary mechanism demanding that each tiny step be an improvement over the previous tiny step, as assessed by natural selection. On the other hand, preassembly creates full or partial genetic wherewithal long before a complex trait makes its worldly appearance. Preassembly allows the so-called “panspermia theory” (that life originated from outer space as espoused by F. Crick of Watson and Crick fame [46]) to become more expendable. Despite remaining uncertainties with preassembly, the theory makes a contribution toward the ultimate goal of having in hand a more complete evolutionary picture.
